# Adoption of E-learning systems: An integration of ISSM and constructivism theories in higher education

**DOI:** 10.1016/j.heliyon.2023.e13014

**Published:** 2023-01-24

**Authors:** Amer Mutrik Sayaf

**Affiliations:** Educational Technology Department, College of Education, University of Bisha, Bisha 61922, Saudi Arabia

**Keywords:** E-Learning system, Interactivity with peers, Information quality, System quality, Users' satisfaction

## Abstract

Based on constructivism theory and the Information System Success Model, this study suggests a research model that examines the factors that influence students' collaborative engagement and satisfaction in an e-learning system setting. This study intends to validate the theoretical concepts and the Information System Success Model (ISSM) on e-learning platforms for user satisfaction and collaborative activity in order to expand the adoption of e-learning systems in higher education. This research used a questionnaire as the primary data collection method to obtain information for the study from 300 responses from different students at the University of Bisha, who all use e-learning platforms. The results were achieved using structural equation modeling, a quantitative research technique (SEM-AMOS). All of the study's hypotheses were supported, according to the findings of the structural model and hypothesis testing. The outcomes of peer interaction (IP) and instructor interaction (IL) have a favorable impact on satisfaction and teamwork, which have a beneficial impact on the usage of e-learning in higher education. This is advantageous for sustainability as well as the adoption of e-learning systems. Finally, the study demonstrates that user happiness and collaborative involvement have a favorable impact on the utilization of e-learning systems. As a result, universities should promote e-learning as a long-term educational strategy.

## 1 Introduction

Prodigious information and communication technology (ICT) advancements have impacted nearly every element of modern life with the Internet's explosive growth. In order to achieve the intended goals and reap the benefits associated with them, the information system (IS) has become deeply ingrained in practically every sector, including businesses, organizations, industries, and the education sector [1,2]. Due to its improved capacity for providing high-quality teaching, the education sector is among those promising and lucrative industries that are most affected by the adoption of technology. However, the adoption level of e-learning affects the e-learning environment [3,4]. The majority of institutions and their managements throughout the world presently rely on the Internet and the IS for their educational activities since the Internet has made it possible for academic operations to be conducted without restrictions regardless of geographical separations [5].

It is believed that the e-learning paradigm represents an expansion of the 1980s-era remote learning model of [6–8]. When it comes to continuing education during the present global lockdown brought on by the coronavirus disease 2019 (COVID-19) pandemic, e-learning has proven to be the only option [9]. All educational institutions throughout the world have made significant investments in e-learning, and many of the courses that were formerly offered only in the traditional classroom setting have been transformed. According to Ref. [10], e-learning and mobile learning are enabling all types of learning, including formal, informal, and non-formal. People are obtaining information through mobile devices in a variety of formats and at a very rapid rate in almost every field. In order to advance the cause of intergenerational education for sustainable development (SD), a pervasive environment for learning at anytime and anywhere has been made available through this technology-assisted learning paradigm [11]. The triple bottom line (TBL), which measures sustainability in three dimensions economic, social, and environmental is used [10]. The United Nations has identified education as one of the five benchmarks for social sustainability [12]. This paradigm will promote social sustainability by offering environmentally friendly ways to learn. In numerous earlier papers, the success of e-learning projects has been investigated [13].

Although research tends to focus more on the perspectives of students, usage of the Internet by both students and teachers has shown that it can change the way that traditional learning methods are used in an engaging online environment [5,14,15]. The reason for this is that both teachers and students can contribute to e-learning platforms. Since it permits access to learning resources without any time or geographic restrictions, the e-learning system (ELS) has started to serve purposes other than instruction [7,16].

As reported in the literature [5,14,15], the teaching and learning systems have undergone amazing changes in the last ten years. An educational approach called collaborative activity places a focus on teamwork between teachers, students, and administrators [17]. It alludes to one of the most efficient teaching strategies and involves methods and settings in which students complete a task on which they depend and are accountable to one another. In a cooperative activity, there may be few or many participants (a small or large group), each with a unique set of skills or IQ levels [[18], [19], [20]]. With the help of this teaching approach, students can participate and communicate their thoughts to the group's other members. The approach encourages positive and productive communication among participants for a more enjoyable learning environment [20].

Making it feasible for people to employ technology in their discussions of subjects, including content, viewpoints, encounters, and technologies, is a primary goal of a collaborative learning system [21]. They are able to stay actively involved and connect their knowledge to the outside world thanks to it. By using e-learning as a platform for education, students can create and share knowledge, improving their social acceptance and self-representation [5]. In this regard, El Mhouti et al. [22] called for the promotion of more effective group learning as well as the comprehension of the interaction between students and teachers with reference to their instruction. Students are receptive to new social media that can aid instructors and students in learning more successfully, despite the fact that they haven't shown much interest in the current channels for connecting with their professors about their training [23]. In light of this, an online collaboration tool and an atmosphere for collaborative activities will support the students' online collaborative efforts. Despite its many advantages, e-learning is not widely employed in developed nations for a number of different reasons.

However, several colleges have switched to e-learning in response to the COVID-19 pandemic in order to maintain their academic programs. In almost every nation, outbreaks of coronaviruses are currently wreaking havoc on the population. Most governments have implemented lockdowns or mobility restrictions to stop the rapid spread of the virus, which has had a significant impact on every aspect of our everyday lives. Regardless of the economy, the education industry has been one of COVID-19's major losses. Traditional teaching strategies have changed as a result. In the event of a pandemic, an e-learning platform can help students and institutions by presenting new opportunities [24]. Only a few studies, including those by Refs. [[25], [26], [27]], have recognized the value of e-learning in high-quality educational offerings. Adopting e-learning has many benefits, including increased parental participation, access to more cutting-edge or novel learning techniques, stronger student motivation, opportunities for self-directed education and adoption, improved ICT device setup, and more [28]. According to empirical data, academicians at nearby HEIs only occasionally use online learning [29,30]. There is a gap in our knowledge of the causes of the resistance to e-learning among academics in neighborhood HEIs, according to a modest body of literature. This makes it possible for academics to look into the factors that influence the adoption of e-learning for long-term educational sustainability at institutions of higher learning where blended learning is now optional.

Students frequently utilize mobile devices and the Internet, and information technology utilization in the education sector is growing. The e-learning system has quickly evolved into a requirement for institutions as a result. The acceptance and adoption of online-based e-learning by students demonstrate their enthusiasm for it. However, researchers found some inconsistencies in their results. Even though mobile services have been added to learning platforms at educational institutions, researchers found that e-learning and student curiosity aren't doing as well as they could [31,32]. A learner's acceptance of and collaborative activity while using eLearning is influenced by a range of factors. Therefore, thorough and varied analysis of these elements is essential [33,34]. As a result, the focus of this study is on how college students wish to use and accept an e-learning system for educational sustainability (ES). According to the study, the COVID-19 pandemic has caused researchers to pay greater attention to e-learning because it is the only way to continue academic pursuits. For students interested in educational sustainability, the fear of COVID-19 has precluded a link between external circumstances and user satisfaction with an e-learning system [35,36].

This study objective to investigate the factors that influence interactive with peers, interactive with lecturers, engagement, perceived technology fit, information quality, system quality, service quality and illustrate the mediating role of users' satisfaction and collaborative activity factor in the relationship between system independent factors and adoption of e-learning systems in higher education. On the other hand, a few study frameworks can forecast a student's BI. This study was motivated by the lack of a framework to predict students' intention to use and adoption of an eLearning system (AE). However, these techniques encourage item reuse rather than facilitating collaborative work carried out through communication and collaboration among writers. This study provides three new insights into how student intent to use an e-learning system for collaborative activity and learner satisfaction can improve e-learning system adoption by (i) identifying variables that affect student collaborative activity and satisfaction to use e-learning for collaborative activity; (ii) investigating relationships between variables; and (iii) making recommendations for future research.

### 1.1 Adoption e-learning system in higher education

The learning procedures at several University of Bisha universities are now being integrated with various software systems, including management learning systems (MLS) and Blackboard [15,37,38]. As a result, information and communication technology (ICT) has enhanced management communications, student-teacher collaboration, interpersonal relationships, and overall educational achievement. Furthermore, a lot of studies have shown how effective e-learning platforms are for delivering distant education. One description of an e-learning system is "the combined use of modern computers and information and communications technology (ICT) to provide teaching, information, and learning content” [39]. An e-learning system is instead characterized as a kind of information system (IS) based on Internet technology that offers the student an infinite number of independent and adaptable teaching and learning opportunities [38,40,41]. The learning processes have been significantly simplified by this technology-based solution [42,43].

Among the crucial components of an e-learning system are benchmarks, the learning environment, learning outcomes, cost-benefit analysis, and ISSM models. A general model assessment of the effectiveness of e-learning programs is important, according to a body of academic research [15,[44], [45], [46]]. Based on information system theory, academics have proposed and evaluated two models: the information system success model (ISSM) and the technology acceptance model (TAM) [15,[44], [45], [46]]. The findings of their research encouraged the establishment of an open-systems paradigm founded on general systems theory, which operates on widely acknowledged concepts and principles with organized and participatory information transmission [[47], [48], [49], [50]].

## 2 Research model and hypotheses

**Fig. 1:**
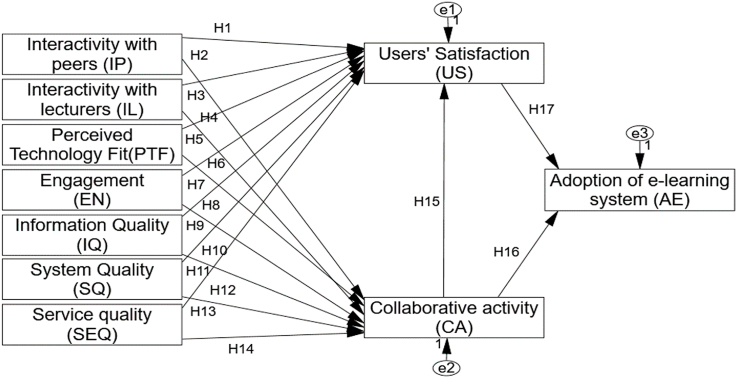
Research model.

Three independent variables system quality, information quality, and service quality are used in the ISSM Model, which is depicted in Fig. 1, to conceptualize the success determinants of information system attributes. Individually or collectively, each of these independent variables influences "learner satisfaction,” and each has an impact on "individual or organization,” which influences how effective an information system is (see Fig. 1). In Ref. [51] proposed an ISSM for measuring IS success in organizations to acquire net benefits. They suggested that IS success is a multifaceted and symbiotic paradigm. Therefore, it is indispensable to study the interrelationships among those dimensions and control them. Subsequently, numerous scholars suggested some reforms to this model [42,48]. Consequently, in Ref. [39] incorporated some of the changes that scholars suggested and accordingly restructured their old model with the updated ISSM, as illustrated in Fig. 1. They decided to augment the dimensions of service quality and user satisfaction. The new model cited service, system, information quality, system use, and user satisfaction as the critical success factors that lead to the adoption of e-learning systems. The researchers contended that if IS success evaluation is desired, then the factors influencing its subsequent use are service, system, information, and quality. User satisfaction and collaborative activity are the outcomes of positive or negative adoptions that will promote the use of IS [52]. Moreover, the research model studies all aspects of constructivism (interaction with peers, interaction with lecturers, engagement, and collaborative activity). So, this learning will utilize constructivism [53,54] to support a fundamental idea: learning is a constructive and active method. Furthermore, our research will use the ISSM pioneered by Refs. [51,53]integrated with constructivism.

### 2.1 Interactive with peer

According to Ref. [55], interaction should be emphasized and studied in all forms of education, whether in-person or online. It is a process that gives students the opportunity to look for new information and develop connections with their teachers, fellow students, and the subject matter of their learning activities [56]. It has been discovered that learning activities play a significant role in shaping students' learning outcomes [57]. The most important component in determining students' satisfaction with online learning and learning results, according to a cross-country survey undertaken by Ref. [57] during the COVID-19 epidemic, was interaction.

Due to technological limitations, interactions in online learning have been noticeably underdeveloped [58], and the importance of interaction has been largely ignored in the literature on distance education [59]. Interaction, which is a crucial part of e-learning systems, has not been properly defined or underlined in the research on remote education, according to Ref. [60]. But according to the research done by Ref. [61], there is more interaction and enjoyment in face-to-face classes than there is in online ones.

Three dimensions of interaction can be distinguished: interaction with teachers, interaction with peers, and interaction with the subject [56]. According to Ref. [62]. According to Ref. [62], regular communication with teachers accounts for 60% of students' satisfaction with online learning, particularly at the beginning of a course.

This is due to the fact that in an online learning environment, instructors must provide each learner with advice, direction, and assistance based on their specific needs; conduct formal and informal evaluations; ensure that learners are making progress; inspire learners; and support learners in applying what they have learned [56,63]. Additionally [64], argued that learner-learner contact in online learning, which enables students to engage, share, and discuss ideas as well as participate in group activities, is crucial for both student enjoyment and academic performance.

Additionally, social interaction with classmates promotes high levels of student satisfaction with a course [65]. In a similar vein, it has been found that contact with the material is highly related to the caliber of the course material, which in turn influences student satisfaction [66]. Learners are more motivated and content when the content is of higher quality [67,68]. On the other hand, a few studies indicated that user satisfaction in several US Massive Open Online Courses was unaffected by learner-learner or learner-instructor interactions [69,70]. In order to create interaction, this study combines these two elements.

H1 Interacting with peer pressure will increase user satisfaction.

H2 Interacting with peer pressure will have a good effect on t Collaborative activity.

### 2.2 Interactive with lecturers

Numerous studies have examined the use of social media and mobile devices in higher education for interacting with peers. 90% of professors [71] use social media for either professional or academic purposes outside of the classroom. The most popular websites for professional outcomes are Facebook and YouTube, with over two-thirds of the teachers using one of these platforms for class sessions and 30% posting content to encourage students to read and study resources [[71], [72], [73]]. Social media and mobile device use in higher education is a relatively new phenomenon with a largely unexplored research area. According to a survey of Economics faculty students at the University of Mortar in Bosnia and Herzegovina [54,71], students are prepared to actively use social networking sites (like SlideShare, etc.) for learning, notably e-learning and communication. Social media is already utilized for information sharing and the sharing of materials. The majority of faculty members utilize social media for professional purposes, share content with distant students, and cooperate using mobile devices and social media, according to a survey by the U.S. Department of Higher Education. A better learning environment is also made possible by the interactive features of online and mobile technology. According to 308 graduate and postgraduate students at University of Bisha, there is a good correlation between online conversation, file sharing, information sharing, entertainment, and learning [74].

H3 User satisfaction will increase as students interact with lecturers.

H4 Collaborative activity will benefit from interaction with the professor.

### 2.3 Engagement

The physical and mental effort that a student puts into activities that are educationally useful has been defined as "student engagement” in all types of education [75,76]. This idea has been connected to various aspects of learning, including completion rates, academic success, and learning satisfaction [77–79]. According to past research [80,81], student involvement is a complex concept with three core substructures: behavioral, emotional, and cognitive engagement. Student behaviors such as attending class and taking part in learning activities while abiding by social and institutional norms are specifically connected with behavioral engagement [82]. "Emotional participation” [83] is the term used to describe students' emotional reactions, both positive and negative, to the educational process and classroom activities. Additionally, learning efforts made by pupils, such as academic self-control and learning tactics or approaches, are referred to as "cognitive engagement” [78,83]. According to Ref. [84], all three aspects of student involvement are interrelated since learning requires students to participate physically (behavioral) as well as psychologically (emotionally and cognitively). Students will be more likely to be unsatisfied with their learning if they don't participate in it in any way [78,85]. Therefore, this study suggests the following:

H5 The influence of student participation will increase user satisfaction.

H6 Student participation will have a good influence on Collaborative activity.

### 2.4 Perceived technology fit

According to Goodhue and Thompson (1995), in terms of task-technology fit (TTF), the properties of a technology are matched with its task features shortly before customers embrace it [86]. Even while people may recognize the benefit of a technology, they will not be able to perform any better if it is not well suited to the work at hand [87]. E-learning solutions are routinely developed to assist users in carrying out a variety of learning-related tasks efficiently [11]. Task-technology fit is essential for examining the acceptance of e-learning by combining many perspectives on the fit based on technology. When establishing the task-technology fit, one might take into account how well a system's operational activities satisfy a person's job requirements [86,88]. The task-technology fit describes the connection between organizational needs, personas, and how well a mobile technology system works [89]. Additionally, the relationship between task-technology fit and the performance criterion has been established, which may be applied in the broader context of evaluating how information technology affects a person's performance [86,88].

H7 The influence of perceived technology fit on user satisfaction will be favorable.

H8 The perception of technology's suitability will have a favorable effect on Collaborative activity.

### 2.5 Information quality

Information quality is a major and critical aspect in evaluating the effectiveness of information and e-learning systems due to the crucial role that information plays in achieving learning objectives and the significant challenges that arise from poor information quality [90]. The relationship between INQ and utilization as well as user satisfaction was examined using the [39] model. Using information systems literature [91], show that there is a strong correlation between information quality and use. Studies by Ref. [92] for knowledge management systems and [93] for health information systems also came to the same conclusion. According to Refs. [94,95], they demonstrated a substantial link between perceived utility, user satisfaction, and information quality in the same setting. Researchers in e-learning have empirically examined the connections between information quality and each of the three notions of usage, satisfaction, and usefulness. For instance Refs. [96,97], discovered a substantial correlation between information quality and both use and satisfaction with the LMS. In Ref. [98] study of e-learning systems in an organizational environment, the association between information quality and perceived utility was shown to be substantial, and [99] found a similar result with web-based LMSs [100].

H9 The influence of information quality will have a favorable effect on user satisfaction.

H10 A favorable impact on collaborative activity will result from information quality fit.

### 2.6 System quality

The LMS platform is the main setting for knowledge transfer in e-learning [100]. As a virtual classroom, it serves to accomplish learning objectives by checking attendance, grading students, and even encouraging student interaction [100]. A few system quality criteria are required [101]. The platform is implemented by the Center for Teaching and Learning at the University and requires Internet compatibility (on Internet Explorer or Google Chrome browsers) in order to be accessed [102]. To use in a performance, the pre-recorded video files are downloaded. The LMS platform's system stability determines how well the downloaded resources display [103,104]. In this study, stability, download speed, and accessibility are considered to be the three most important aspects of a good system. These system features are used in the ISSM Model in order to investigate the connection between system attributes and learner satisfaction [105]. This study makes the supposition that if the system requirements are met, learner satisfaction will increase.

H11 The influence of system quality will have a favorable effect on user satisfaction.

H12 The influence of system quality fit will have a favorable impact on collaborative activity.

### 2.7 Services quality

The DeLone and McLean model was updated to include this additional architecture [51]. The DeLone and McLean model [106], which assumed direct correlations between service quality and both utilization and user satisfaction in their model, is related to the significance of this construct as a measure of information systems performance. The information systems field has used the construct. For instance Ref. [105], in an online buying system established the link between SRQ and satisfaction. According to Ref. [107], the direct correlation between SRQ and use in an e-government system is considerable. Similar to this, the association between SRQ and satisfaction in the context of e-learning was found to be significant in the [108]models. In the study conducted by Ref. [109], it was demonstrated empirically that the association between SRQ and perceived usefulness provided in the conceptual model created by Ref. [110]was substantial.

H13 The influence of service quality will increase user satisfaction.

H14 The effectiveness of the services' quality will have a favorable effect on collaborative activity.

### 2.8 Collaborative activity

To make up for the loss of interaction in e-learning, collaborative activity is a sort of engagement and socialization process that encourages involvement, interaction, and communication, whether in a virtual community or in person [103]. In this study, the instructor assigns specific assignments or projects to groups of students [103]. Each group works on tasks independently, with support from one another, and shares and evaluates the results of the assignments among group members [111]. These activities are necessary to foster dedication, guarantee higher thinking in learners, and promote long-term growth [54]. The instructor's leadership in the students' real commitment will have a significant impact on having a meaningful educational experience, even though it is a little less regimented but still an important component of learning [112]. Students improve their ability to communicate with their peers to solve difficulties or plan social events as a result of the online learning environment [22,113]. In order to establish collaborative learning through online learning in higher education, there are some essential conditions that need to be addressed. The development of active collaborative learning and the encouragement of cognitive abilities such as reflection and metacognition serve as representations of these situations [113,114]. According to some researchers, including [115], the use of online learning by students to complete their assignments has a favorable impact on their level of learning.

H14: User satisfaction will increase as a result of collaborative activities.

### 2.9 User satisfaction

The cognitive congruence between user satisfaction and website quality is known as satisfaction [115]. According to Ref. [116] study on e-learning courses, the satisfaction of the class is strongly connected with service quality, system quality, and particularly information quality. According to e-learning studies [117], individual variances in learning preferences can affect satisfaction. Our main concern is whether students in the context of higher education are content with the outcomes of e-learning, as was already mentioned. Therefore, a variety of variables may have an impact on how satisfied the students are. This idea is supported by studies on student satisfaction with e-learning outcomes [118] and earlier investigations of the theory behind what motivates student-student interaction, efficient help, learning resources, and the learning environment [119]. According to Ref. [120], there are six factors that affect how enjoyable e-learning is perceived. Similar to this [121], asserted that interactive learning environments, perceived self-efficacy, and felt concern may have an impact on reported satisfaction. Users' satisfaction with using an e-learning system has been reported to have a substantial impact on users' intention to use an e-learning system, which in turn has a big impact on the quality of the system, the information, and the service [121]. Thus, we infer that a number of potential factors may have an impact on how satisfied users are with their e-learning experience.

H15 The acceptance of e-learning will be positively influenced by user satisfaction.

## 3 Research methodology

Almost 350 university students were surveyed; a total of 300 questionnaires were returned, resulting in an 85.7% response rate. 50 questionnaires were found to be missing information after manual screening; they were thus disqualified. In support of such exclusions [122], argued that outliers must be considered since they might cause incorrect statistical inferences. As a result, 300 questionnaires in total were reliable and valid, and the learning system has been promoted by several institutions, including those in Saudi Arabia. As a result, the purpose of this study is to use empirical research to construct a model for measuring the adoption of an e-learning system (AE). The study's sample comprised students with both undergraduate and graduate degrees who utilized e-learning. Demographic data was requested from the respondents in the first part. The second segment, which contained 50 items, was devoted to measuring the research model's components. A five-point Likert scale was utilized for items including ISSM and constructivism theories, model constructs, and demographic data, with one indicating strong disagreement and five suggesting strong agreement. The measurement model's validity and reliability were evaluated using Amos version 23 and SPSS Statistics version 26. Table 1 reveals that 109 (36.3%) were female and 191 (63.7%) were male. In addition, 34 respondents (11.3%) were between the ages of 18 and 22, 86 respondents (28.7%) were between the ages of 23 and 28, 124 respondents (41.3%) were between the ages of 29 and 34, 46 respondents (15.3%) were between the ages of 35 and 40, and 10 respondents (3.3%) were over the age of 41. For the model's goodness of fit, factor loadings were utilized to establish build validity, composite reliability, Cronbach's, and convergence validity, as stated by Jung et al. (2008). Cronbach's was found to be 0.920 based on standardized items. The reliability coefficient (Cronbach's) for final test designs is shown in Table 4; all variables were found to be appropriate. For more details, see Table 4.

**Table 1:** Demographic profile.

Items	Description	N	%
Gender	Male	191	63.7
	Female	109	36.3
Age	18–22	34	11.3
	23–28	86	28.7
	29–34	124	41.3
	35–40	46	15.3
	41– Above	10	3.3
Specialization	Science &Technology	150	50.0
	Social Science	81	27.0
	Management	49	16.3
	Others	20	6.7

**Table 2:** Records of the good features of the measurement model.

Model	χ2/*df*	CFI	TLI	SRMR	RMSEA
*Target*	*≤ 5.0*	*≥ 0.90*	*≥ 0.90*	*≤ 0.09*	*≤ 0.08*
Model 1 (Final model)	2.52	0.925	0.914	0.048	0.061

**Table 3:** Validity and reliability.

	IL	PTF	EN	IP	SEQ	SQ	IQ	CA	US
IL	0.857								
PTF	0.345	0.762							
EN	0.238	0.442	0.783						
IP	0.584	0.261	0.222	0.832					
SEQ	0.347	0.350	0.295	0.260	0.710				
SQ	0.577	0.383	0.354	0.563	0.361	0.834			
IQ	0.585	0.303	0.270	0.547	0.321	0.580	0.844		
CA	0.639	0.332	0.193	0.609	0.319	0.606	0.588	0.899	
US	0.613	0.381	0.314	0.610	0.279	0.631	0.578	0.623	0.843
AE	0.564	0.320	0.207	0.514	0.355	0.582	0.647	0.574	0.578

**Table 4:** Load, CR, AVE, alpha.

Construct	Items	Load	CR	AVE	Alpha
Interactive with peer	IP_1	0.875	0.913	0.679	0.911
	IP_2	0.832			
	IP_3	0.690			
	IP_4	0.882			
	IP_5	0.826			
Interactive with lecturers	IL_1	0.850	0.914	0.680	0.912
	IL_2	0.839			
	IL_3	0.851			
	IL_4	0.829			
	IL_5	0.750			
Perceived technology fit	PTF_1	0.795	0.895	0.631	0.895
	PTF_2	0.819			
	PTF_3	0.833			
	PTF_4	0.751			
	PTF_5	0.772			
Engagement	EN_1	0.782	0.895	0.632	0.895
	EN_2	0.798			
	EN_3	0.846			
	EN_4	0.784			
	EN_5	0.760			
Information quality	IQ_1	0.844	0.925	0.713	0.925
	IQ_2	0.851			
	IQ_3	0.843			
	IQ_4	0.860			
	IQ_5	0.822			
System quality	SQ_1	0.819	0.912	0.674	0.909
	SQ_2	0.825			
	SQ_3	0.827			
	SQ_4	0.828			
	SQ_5	0.805			
Services quality	SEQ_1	0.892	0.912	0.677	0.908
	SEQ_2	0.902			
	SEQ_3	0.822			
	SEQ_4	0.630			
	SEQ_5	0.837			
Collaborative activity	CA_1	0.872	0.922	0.703	0.920
	CA_2	0.817			
	CA_3	0.777			
	CA_4	0.830			
	CA_5	0.891			
User satisfaction	US_1	0.789	0.899	0.639	0.897
	US_2	0.818			
	US_3	0.804			
	US_4	0.814			
	US_5	0.772			
Adoption of e-learning	AE_1	0.862	0.906	0.926	0.722
	AE_2	0.876			
	AE_3	0.873			
	AE_4	0.797			
	AE_5	0.838			

### 3.1 Data collection analysis

300 undergraduate and graduate students (local and international) at the University of University of Bisha responded to a structured questionnaire using a 5-point Likert scale to gather the results. Based on these analyses, the sample size of this study (N = 300) is acceptable according to Krejcie and Morgan (1970). A structured physical survey was used to collect data from students at University of Bisha in Saudi Arabia to test the theoretically developed model. The sample size was determined by using the following formula:

$$SS=\frac{x^{2}\left(p\right)\left(q\right)}{e^{2}}$$

where SS = Sample Size; Z = 1.96 (95% confidence level); P = prevalence level (0.5 used for sample size needed); Q = (1 − p); E = error term (0.05). By inserting values into the formula, the sample size would be:

$$SS=\frac{1.69^{2}\left(0.50\right)\left(0.50\right)}{0.05^{2}}$$

$$SS=\frac{2.8561\left(0.25\right)}{0.0025}$$

$$SS=\frac{0.7140}{0.0025}$$

SS=285.6

The COVID-19 situation restricting physical movement necessitated administering the questionnaires online via emails and Google Form links. Both undergraduate and graduate students received an online version of the questionnaire. User satisfaction and collaborative engagement were affected by the interconnected components of e-learning systems. Therefore, each and every one of the variables satisfies the Cronbach alpha coefficient, which ranges from 0.70 to 0.90. Cronbach's reliability coefficient, which is 0.920, is examined in the reliability analysis. The inter-construct correlations linked to the variable (IC) had to be less than 0.80, the AVE rate had to be greater than or equal to 0.5, and the AVE square had to be higher [123]. These three criteria were used to assess discriminant validity. Additionally, loadings of confirming factors of 0.7 and higher were discovered. Cronbach's alpha scores of 0.70 or above and composite dependability were deemed acceptable [122].

### 3.2 Measurement model analysis

For university students, 300 sample questionnaires were given out. All of them have proven to be helpful. The construction components confirmed that prior investigations had supported the material validity of the measurement scales. The survey form that was chosen was as follows: Interactivity with peers and lecturers was adopted from Refs. [124,125]; perceived technology fit from Ref. [126]; engagement from Refs. [127,128]; information quality from Refs. [7,11,129]; system quality from Refs. [7,129]; service quality from Refs. [7,11,129]; users' satisfaction from Refs. [7,11,130]; collaborative activity from Ref. [131]. In the end, adoption of e-learning systems was adopted from Refs. [7,132] and any permissible outside loading.

## 4 Data analysis

### 4.1 Measurement model and validity and reliability analysis

**Fig. 2:**
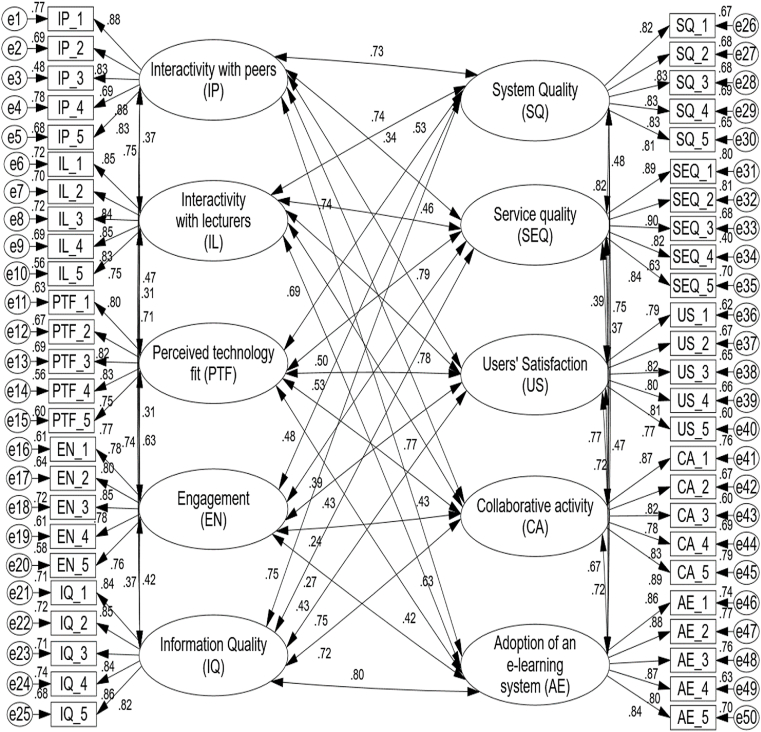
Outcomes of the proposed model for all response groups.

SEM was used in this work as a key statistical method in AMOS 23 to examine the outcomes depending on CFA. Over convergence was investigated using this model [123]. Additionally, according to Ref. [122], "goodness-of-fit” techniques such as standard chi-square, chi-square, RFI, TLI, and the IFI, the model fits well when the CFI value is greater than or equal to 0.90. Additionally, as shown in Table 2, where "RMR” is acceptable, the "RMSEA” complies with the recommended standard of less than or equal to.08 to support the needed suit [122]. The model's suitability indices, specifically, are AVE and CA. To satisfy all parameters, CR values between 0.895 and 0.926 are acceptable, as are CA values between 0.895 and 0.928. Furthermore, the AVE varied between 0.631 and 0.722, exceeding the anticipated value of 0.50 (see Table 4). This suggests that all of the loading factors are significant and above the threshold of 0.50, satisfying the presented correlations [122,123] and measuring the independent, mediator, and dependent variables mentioned in Fig. 2. These variables are measured in Table 3 and are independent, mediator, and dependent variables.

### 4.2 Structural equation model analysis

**Fig. 3:**
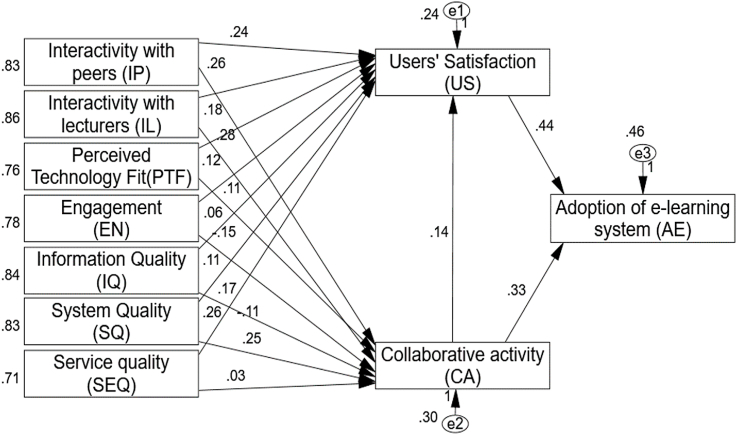
Results for the proposed model.

The route modeling analysis was used to investigate how peer interaction, lecture interaction, and engagement factors influenced the use of e-learning systems, as well as constructivism and ISSM as perceived technology fit, information quality, system quality, and service quality factors on e-learning utilization for user satisfaction through collaborative activity. The results are given and evaluated in light of the results of the hypothesis testing. The structural equation model was examined by the authors using CFA in the next step of the process. Because all of the assumptions established between the fifteen fundamental components, or hypotheses, were accepted, Fig. 3 shows the structural model. The structural model is presented in Table 5, which demonstrates that the model's key statistics are quite robust, indicating applicability and a useful model for verifying the assumptions. All initial hypotheses were supported by the study's findings, which show that e-learning positively affects the adoption model in higher education. The results also support theories concerning the direction of the interaction between the structural model and the variables in the model. The un-standardized coefficients and standard errors of the structural model are presented in Table 4. Fig. 3 depicts all of the hypotheses between the seventeen key constructs; fifteen of them were accepted, and only two were rejected: "no engagement between users for CA (0.06-H7), and "no service quality for CA” (0.03-H14), "IP have on e-learning system having user satisfaction (0.24-H1) and collaborative activity (0.26-H2), "IL have on e-learning system having user satisfaction (0.24-H (0.15-H8), IQ is related to US with an e-learning system (0.11-H9) and collaborative activity (0.17-H10), SQ is related to US with an e-learning system (0.26-H11) and collaborative activity (0.25-H12), SEQ is related to US with an e-learning system (0.26-H13), collaborative activity is related to US with an e-learning system (0.14-H15), and adoption of an e-learning system (0 (0.44-H17). In addition, Fig. 3 and Table 5 illustrate the path coefficient and loading value of the path lines inside the Amos. Only two of the fifteen hypotheses that were put forth for this study were rejected. In detail, as proposed for the relationships between interactivity with peers and users' satisfaction (H1) (β = 0.236, t = 4.760), and Collaborative activity (H2) (β = 0.257; t = 4.784), the hypotheses are supported. For the relationship between interactivity with lecturers and users' satisfaction (H3) (β = 0.178; t = 3.434), and Collaborative activity (H4) (β = 0.277; t = 4.937), the hypotheses are supported. The hypotheses H5 and H6 is also supported where perceived technology-fit is significantly predicted by users' satisfaction (β = 0.115; t = 2.637) and collaborative activity (β = 0.105; t = 2.162). Moreover, the significant role of engagement and user satisfaction (H7) is also reported (β = 0.064; t = 1.558) and the hypothesis isn't accepted. In the same way, the results for engagement and collaborative activity (H8) (β = −0.150, t = −3.287) indicate support. For the relationship between information quality with users' satisfaction (H9) (β = 0.111 t = 2.267) and information quality with collaborative activity (H10) (β = 0.167, t = 3.103) are accepted. For the hypotheses H11 and H12, the direct effect of system quality on users' satisfaction (β = 0.263, t = 4.933) and system quality on collaborative activity (β = 0.248, t = 4.258), the hypotheses are accepted. Moreover, the significant role of service quality and user satisfaction (H13) is also reported (β = −0.111; t = −2.697) and the hypothesis is accepted. In the same way, the results for service quality and collaborative activity (H14) (β = 0.028, t = 0.597) indicate Unsupported. Furthermore, results for collaborative activity to users' satisfaction and Adoption of e-learning system (H15, H16), (β = 0.140, t = 2.713) and (β = 0.334, t = 5.634) indicate support for the hypotheses. Finally, users' satisfaction is also informed to be a significant predictor for Adoption of e-learning system for educational sustainability (H17) (β = 0.438, t = 7.163); the hypothesis is supported.

**Table 5:** Hypothesis testing results of structural model.

H	Factors	Estimate	S.E.	C.R.	*P*-value	Results
H1	IP------ > US	0.236	0.050	4.760	0.000	Accepted
H2	IP------ > CA	0.257	0.054	4.784	0.000	Accepted
H3	IL ------ > US	0.178	0.052	3.434	0.000	Accepted
H4	IL ------ > CA	0.277	0.056	4.937	0.000	Accepted
H5	PTF------ > US	0.115	0.044	2.637	0.008	Accepted
H6	PTF------ > CA	0.105	0.049	2.162	0.031	Accepted
H7	EN------ > US	0.064	0.041	1.558	0.119	Rejected
H8	EN------ > CA	−0.150	0.046	−3.287	0.001	Accepted
H9	IQ------ > US	0.111	0.049	2.267	0.023	Accepted
H10	IQ ------ > CA	0.167	0.054	3.103	0.002	Accepted
H11	SQ ------ > US	0.263	0.053	4.933	0.000	Accepted
H12	SQ ------ > CA	0.248	0.058	4.258	0.000	Accepted
H13	SEQ ------ > US	−0.111	0.041	−2.697	0.007	Accepted
**H14**	SEQ ------ > CA	0.028	0.046	0.597	0.550	Rejected
H15	CA ------ > US	0.140	0.051	2.713	0.007	Accepted
**H16**	CA ------ > AE	0.334	0.059	5.634	0.000	Accepted
**H17**	US ------ > AE	0.438	0.061	7.163	0.000	Accepted

## 5 Discussion and implication

By creating a research model centered on the function of collaborative activity as a source of sustainability in higher education, this study enhanced our theoretical understanding of how to employ e-learning as a source of educational sustainability. This study demonstrates the beneficial relationships between the characteristics of the learning environment and learner retention. It also highlights the link between learner satisfaction and the instructor's participation in the online learning environment. This is one of the first studies to look at how e-learning is used for teaching and learning in higher education at University of Bisha using constructivism theory and the ISSM paradigm. Users' satisfaction and collaborative activity learning are significantly influenced by their interactions with peers, lectures, engagement, perceived technological fit, information quality, system quality, and service quality (see Fig. 3). The adoption of e-learning systems for long-term educational sustainability was impacted by the degree of user happiness and collaborative participation. As a result, the findings supported the established hypotheses and the design of the research methodology. The findings demonstrate that collaborative involvement had an effect on users' satisfaction with the usage of e-learning tools for teaching and learning in higher education. To put it another way, before university students and instructors will adopt e-learning for long-term educational sustainability, they must see proof of collaborative activity and user satisfaction. E-learning ought to be widely adopted and offer straightforward instructions. The results also showed how crucial it is for instructors to explain how students should use online learning to study course material because users' acceptance of online learning for long-term educational sustainability grows with their satisfaction with it. In order to examine the factors influencing the adoption of an e-learning system for teaching and learning in higher education, this study included the ISSM model, which successfully explained the adoption practice of e-learning during COVID-19 as perceived by students in higher education from students' and lecturers' universities. The research model takes into account the implementation of e-learning for teaching and learning in higher education as well as the constructivism theory parts IP, IL, PTF, EN, IQ, SQ, SEQ, US, and CA. Therefore, the study model determines that constructivism theory variables and the ISSM model have the biggest impact on user satisfaction and adoption of e-learning systems for educational institutions when used as a teaching and learning tool.

The results show that peer interaction plays a key role in learning, supporting hypotheses H1 and H2, and showing that IP has a positive impact on users' satisfaction with and involvement in collaborative activities related to the use of e-learning in education. To put it another way, the high IP factor and appropriateness of the e-learning system encourage user satisfaction and collaborative learning. The benefits of e-learning and the main intellectual property that goes with it have been extensively studied by academics. The results of this investigation thus support earlier findings [71,133]. The results of the study provide significant evidence for the interactivity with lectures variable, confirming hypotheses H3 and H4 that interactivity with lectures has a favorable effect on users' satisfaction and collaborative learning. Or, to put it another way, when an e-learning system is acceptable and simple to use, the higher interactivity with lectures contributes to improved user satisfaction of the e-learning system in education and subsequently increased collaborative engagement.

The advantages of interactive lectures in the context of online learning have been examined by several academics. The study's findings support previous connections between factors [71,133,134]. Therefore, using e-learning to connect with mentors and peers leads to improved user happiness and collaborative activity, which in turn affects e-learning adoption. The higher education authority should offer a space where students can develop their intellectual abilities. According to the empirical study, communication devices let students recover information and communicate with others in real-time regarding sharing the contents of learning materials, which increases student satisfaction. Additionally, such advanced communication tools would be more beneficial for students who are uncomfortable speaking in front of their peers; teachers might become more accessible online for collaborative learning and teaching in a global setting [10].

The study's findings also validate Hypotheses H5 and H6, providing strong evidence that the perceived technology-fit (PTF) component has a positive effect on users' satisfaction and collaborative engagement. When e-learning is desirable and appropriate in educational institutions, a greater perceived technology-fit factor contributes to increased user enjoyment and group participation.

According to the results, a number of dimensions, including perceived technology fit, showed a favorable and substantial link with students' happiness, indicating a rise in their usage intentions and an impact on their performance. These studies' findings about the important benefits of perceived technological fit, user pleasure, and collaborative engagement are supported by earlier research. Thus, it can be concluded that before choosing to use e-learning, students assess its suitability for fulfilling their academic needs and its relevance to their education. Thus, the outcomes of this study support previous findings of numerous connections [36,126,135].

The next parameter is engagement; the results of the study did not support the relationship between engagement and the users' satisfaction variable, supporting hypothesis (H7) that engagement does not have a beneficial effect on users' contentment. These findings, however, did not agree with those of the earlier studies [71,136]. Furthermore, the study's results substantially support the engagement variable, confirming hypothesis (H8) [127] and showing that engagement influences collaborative activity for teaching and learning in higher education positively [54,71].

The students' high levels of involvement, contentment, and collaborative activity further suggested that they valued the benefits of their online education during the epidemic. The students expressed agreement that they had a strong internal motivation for and favorable views regarding their online learning, and that they actively engaged in it [54,71].

To put it another way, the growing use of an e-learning system as a collaborative activity in education is a result of increased engagement with and acceptance of the system. Numerous scholars have looked into the importance of involvement in the area of e-learning. The results of this investigation thus lend support to past studies [54,71,137].

The study's findings also show a strong relationship between information quality with user satisfaction and collaborative engagement for e-learning use as educational sustainability, lending support to the hypotheses (H9 and H10).

This demonstrates that user satisfaction and collaborative engagement are influenced by the quality of the information available. Information quality factors such as providing students with sufficient and required information, concise and clear information, updated content, and an attractive design of the content are important for students to enjoy and enjoy their experience with e-learning and contribute to their overall satisfaction. Students can complete their learning assignments more rapidly thanks to the e-learning system's organization of the content and information into logical and understandable components [54,71].

Moreover, the increased information quality encourages continued use of the system as a long-lasting educational tool, demonstrating that technology is appropriate when an e-learning system influences information quality and is deemed acceptable. Prior studies have looked into the importance of information quality in the context of e-learning. As a result, the findings of this study support prior connections between variables [11]. These findings, however, did not agree with those of the earlier studies [7].

Additionally, this study's results significantly support the system quality variable, confirming hypotheses (H11 and H12), demonstrating that system quality affects users' satisfaction and collaborative activity for e-learning use as a sustainable educational practice.

Our research shows that students view an e-learning system's usefulness in providing helpful functions for efficient learning when it offers high-quality functions to achieve learning goals and tasks and facilitate the learning process. Additionally, if comparable capabilities are offered, students may constantly access course materials, interact with their peers, and communicate with instructors, they will view the system as valuable. According to Ref. [138], these elements lead to students' happiness and collaborative activity with the system and improve their willingness to use the system.

In the context of e-learning, previous studies have emphasized the significance of system quality. Thus, the findings of this study support earlier conclusions [7,11] about the connections between numerous parameters. The findings categorically support hypothesis (H13) and demonstrate that student's satisfaction is positively impacted by student involvement in educational institutions. They also clearly support the service quality component. On the other hand, hypothesis (H13) shows that user satisfaction and using the e-learning system have a relationship.

Our research suggests that students' happiness and collaborative activity with the e-learning system enhance if they obtain appropriate technical support services from a help desk or technical staff. Students who receive high-quality technical support from technical staff have a higher perception of the value of the e-learning system, which improves their use of it.

The results demonstrate a substantial and positive association, supporting the premise. The results of this investigation thus support earlier findings of various correlations [11]. While the study's results disproved the relationship between service quality and collaborative activity, they didn't support Hypothesis (H14), which states that service quality doesn't have a beneficial influence on collaborative activity. In other words, students' perceptions of the collaborative nature of the e-learning system and the system's use are unaffected by the quality of services provided to them by IT employees.

These findings, however, did not agree with those of the earlier studies [7,71]. Both H15 and H16 were positively correlated with the collaborative activity, with the adoption of e-learning platforms and user satisfaction having greater effects. When an e-learning system improves collaborative activity, user satisfaction and e-learning system adoption rise in direct proportion. Several studies have looked at the importance of teamwork in the world of online learning. As a result, using e-learning for group projects that satisfy mentors and peers leads to improved student engagement, which in turn influences how readily students embrace e-learning systems. The higher education authority should offer a space where students can develop their intellectual abilities. According to the empirical study, it can be concluded that engaging in collaborative activities helps students find information and communicate with others about the contents of educational materials in real-time.

Therefore, this study's findings are consistent with earlier ones [103,139]. Last but not least, the study's results show that the users' satisfaction variable has a substantial correlation with the adoption of e-learning systems in schools, confirming Hypothesis (17). To put it another way, the more users who are satisfied with an e-learning system, the more likely it is to be used in educational settings. Numerous academics have looked into the value of user satisfaction in the context of e-learning. As a result, the findings of this study support earlier connections between variables [7,140].

### 5.1 Theoretical and practical implications

The first contribution of this research is the creation of a multi-dimensional, all-inclusive model for assessing the effectiveness of e-learning. The model was created based on a thorough assessment of the literature and consideration of four methods for determining if e-learning is successful: the constructivism theory and the ISSM model. Because various viewpoints have been taken into account in relation to various aspects of interaction with peers and lecturers, perceived technology fit, engagement, information quality, system quality, service quality, users' satisfaction, collaborative activity, and adoption of e-learning systems, and because these encompass the key elements of the existing approaches, this new model is believed to be comprehensive.

Second contribution: this study went a step further and provided an empirical analysis of the model created by including the variables that affect the uptake of e-learning systems. Interactivity with peers and lecturers, perceived technological fit, engagement, content quality, system quality, and service quality are the seven types of criteria postulated and empirically explored as antecedents of users' satisfaction, collaborative activity, and adoption of e-learning systems. The discovery of e-learning success variables is the second contribution of this research, and all of these components taken as a whole are valid and significant metrics.

The third contribution of this work, however, is that it is one of the first to provide a thorough characterization of e-learning success variables and experimentally evaluate the correlations between the measures in a single model. The performance of the developed model is the focus of the fourth contribution. The model demonstrated a substantial mediating predictive power for the use of e-learning between collaborative activity and user pleasure. The final contribution, the study, offers significant theoretical advancements for constructivism and the ISSM. By suggesting an expansion of the ISSM model literature and constructivism theories, it contributes to those fields of study. This study also supports the validity of the ISSM model for assessing the effectiveness of e-learning programs in Saudi Arabia. Therefore, this study differs from earlier studies in the following ways: The first implication relates to the importance of established structures. The positive association between constructivism aspects like interaction with peers and lecturers, engagement, and the ISSM model's information quality, system quality, and service quality is particularly crucial for user satisfaction and collaborative activity. Since e-learning should be viewed as simple and advantageous, universities may use technology to explain how to use it by providing instructional tools to help students and lecturers understand how to use it. Third, it's important to educate students about the many benefits of technology, such as how it can be used to deliver course content or accomplish other long-term learning goals. As a result, students will be more motivated to use and implement e-learning for the long-term success of education. Although this research demonstrates that there is statistical support, it has a number of drawbacks. Since all of the respondents in this sample are from the same university, more participants from different majors would be required in future studies. Since the sample lacked qualitative evidence, it was founded on students' expectations, which might not coincide with teachers' perspectives. It is suggested that follow-up studies be conducted in other countries.

## 6 Conclusion and future work

E-learning is expected to become a common method of instruction and learning in higher education. Given the system's importance in promoting globalization and regional integration, developing economies, which strive to catch up with their counterparts in advanced economies, are currently stepping up their adoption and/or deployment of e-learning systems. In order to create effective adoption and/or implementation policies, governments and policymakers in emerging economies must be aware of the relevant considerations. This study looked into the crucial factors that influence users' satisfaction and cooperative behavior among distance learning students at University of Bisha. The constructivism theory and information systems success model served as the study's guiding principles. A survey instrument was filled with 300 valid replies from University of Bisha distance education users after questionnaires were distributed to university students.

This research used Version Amos 23 of the Amos paradigm to process and examine the information obtained from the surveys. The measurement and structural models underwent evaluations. In answer to the research questions, we find that, first, peer interaction, interactive lectures, information quality, system quality, and service quality are the characteristics that influence users' satisfaction and collaborative behavior of distance learners. Second, according to our findings, a strong predictor of learning outcomes for distant learners in an online learning environment is contentment. The links between determinants of users' satisfaction and the collaborative activity of distance learning students at University of Bisha were shown to be mediated by users' satisfaction and activity. It is crucial to maximize educational institutions' e-learning expenditures because it was discovered that users' satisfaction and collaborative engagement were efficient mediators of the relationship between each of the instructor characteristics and the adoption of an e-learning system by distance learning students. It is crucial to examine different contexts and settings for qualities that could influence how e-learning is employed during epidemics like COVID-19. Even though this research reveals meaningful information regarding factors affecting collaborative activity and user satisfaction with e-learning by students, Still, some limitations exist, like the fact that data was collected from students of King Saud University only. It is advised that the authors repeat their research in other provinces other than Saudi Arabia, which has a different ecology, and take these limitations into consideration. The business of respondents also affects the study, and the variables used may be subject to change over time, so longitudinal studies can give more generalized results. In the future, more external variables could be added to examine their impact on the ISSM and constructivism theories, and the effect of demographic variables on the ISSM and constructivism theories in the Saudi Arabian context could also be studied.

## Author contribution statement

Amer Mutrik Sayaf, Ph.D: Conceived and designed the experiments; Performed the experiments; Analyzed and interpreted the data; Contributed reagents, materials, analysis tools or data; Wrote the paper.

## Data availability statement

Data included in article/supp. material/referenced in article.

## Declaration of interest’s statement

The authors declare no conflict of interest.
