# Supplementary material for: Adoption of E-learning systems: An integration of ISSM and constructivism theories in higher education
Source: Heliyon. 2023 Jan 24;9(2):e13014. doi: 10.1016/j.heliyon.2023.e13014 (PMC9937898; doi:10.1016/j.heliyon.2023.e13014)
Supplement: Multimedia component 1 [file mmc1.docx]

Appendix A

| **Interactive with peer (IP)** | | |
| --- | --- | --- |
| 1. | | E-learning facilitate interaction with peers |
| 2. | | E-learning gives me the opportunity to discuss with peers. |
| 3. | | E-learning allows the exchange of information with peers. |
| 4. | | Using E-learning increase my knowledge sharing capabilities. |
| 5. | | Using E-learning improve my communication skills with research members and peers. |
| **Interactive with lecturers (IL)** | | |
| 6. | | E-learning facilitate Interactive with lecturers |
| 7. | | E-learning gives me the opportunity to discuss with lecturers. |
| 8. | | E-learning allows the exchange of information with lecturers. |
| 9. | | Using E-learning in my study facilitates discussion with research group members, peers and lecturers. |
| 10. | | I use E-learning for getting resources from my lecturers and supervisors. |
| **Perceived technology fit (PTF)** | | |
| 11. | I think that using e-learning is well suited for the way to learn. | |
| 12. | E-learning is a good tool to provide the way I like to study tasks. | |
| 13. | Using e-learning fits well for the way I like to study tasks. | |
| 14. | I think that using e-learning would be a good way to learn. | |
| 15. | E-learning functions are suitable for helping me complete my coursework. | |
| **Engagement (EN)** | | |
| 16. | By using e-learning I engage in interactions with my peers | |
| 17. | By using e-learning I engage in interactions with my lecturers. | |
| 18. | By using e-learning I learned how to work with others effectively. | |
| 19. | By using e-learning I am satisfied with the engagement in my studies. | |
| 20. | By using e-learning in my study, my group members, peers and faculty interactions made me feel valuable. | |
| **Information Quality (IQ)** | | |
| 21. | E-learning provides information that is relevant to my needs. | |
| 22. | E-learning provides comprehensive information. | |
| 23. | E-learning provides information that is exactly what I want. | |
| 24. | E-learning provides me with organized content and information. | |
| 25. | E-learning provides up to date content and information. | |
| **System Quality (SQ)** | | |
| 26. | E-learning provides a proper online assistance and explanation. | |
| 27. | E-learning is aesthetically satisfying. | |
| 28. | E-learning optimizes response time. | |
| 29. | E-learning is user friendly. | |
| 30. | E-learning provides interactive features between users and system. | |
| **Service Quality (SEQ)** | | |
| 31 | Overall, the level of service quality I received from the E-learning system during the class was good. | |
| 32. | Overall, the level of service quality I received from the E-learning system during the class was excellent. | |
| 33. | Overall, the level of service quality I received from the E-learning system during the class was high. | |
| 34. | E-learning provides a proper online assistance and explanation. | |
| 35. | There are enough and clear instructions/training about how to use E-learning. | |
| **Collaborative activity ( CA)** | | |
| 31 | I felt that I actively collaborated in my experience. | |
| 32. | I felt that I have co-created my own experience. | |
| 33. | I felt that I had free reign to co-create my own experience. | |
| 34. | I am satisfied with active collaboration in my class. | |
| 35. | I think that collaborative learning with using of E-learning increases my  Understanding of how to perform tasks. | |
| **Users satisfaction (TTF)** | | |
| 36 | I enjoy the experience of using E-learning with peers. | |
| 37. | I enjoy the experience of using E-learning with lecturers. | |
| 38. | I am satisfied with using E-learning for learning. | |
| 39. | I am satisfied with using E-learning to improve my studies. | |
| 40. | Overall, I am pleased with the experience of using E-learning. | |
| **Adoption of E-Learning (AE)** | | |
| 41 | Using E-learning has increased my knowledge and helped me to be successful. | |
| 42. | E-learning is a very effective educational tool and has helped me to improve my learning process. | |
| 43. | E-learning makes communication easier with the instructor and other classmates. | |
| 44. | E-learning saves my time in searching for materials and cuts down expenditure such as paper cost. | |
| 45. | I have control over the E-learning system on campus. | |
